# Neuroimmune alterations and nociceptive behavior in oral carcinogenesis in rats

**DOI:** 10.1111/eos.70107

**Published:** 2026-05-14

**Authors:** Joana Maria dos Santos Alves, Luiza Clertiani Vieira Alves, Vitória Tavares Bessa, Anamaria Falcão Pereira, Roberto César Pereira Lima Júnior, Mariana Lima Vale, Karuza Maria Alves Pereira, Delane Viana Gondim

**Affiliations:** ^1^ Postgraduate Program in Dentistry Faculty of Pharmacy Dentistry and Nursing Federal University of Ceará Fortaleza Ceará Brazil; ^2^ Dentistry Course Faculty of Pharmacy Dentistry and Nursing Federal University of Ceará Fortaleza Ceará Brazil; ^3^ Postgraduate Program in Pharmacology Faculty of Medicine Federal University of Ceará Fortaleza Ceará Brazil

**Keywords:** 4‐nitroquinoline 1‐oxide, glial cells, macrophage, oral cancer, oral carcinogenesis, pain, trigeminal pathway

## Abstract

Oral cancer pain is a frequent and debilitating symptom associated with tumor progression. Although neuronal mechanisms have been extensively studied, the role of immune and glial cells in the trigeminal nociceptive pathway remains poorly understood. In this study, male Wistar rats were exposed to 4‐nitroquinoline‐1‐oxide for 8, 12, 16, or 20 wk. Mechanical allodynia was assessed using the von Frey test, and immunohistochemistry/immunofluorescence analyzed macrophage infiltration in the tongue and activation markers (nitrotyrosine, p‐p38, glutamine synthetase, and Iba‐1) in the trigeminal ganglion (TG) and spinal trigeminal subnucleus caudalis (Sp5C), focusing on animals with the highest nociceptive responses and macrophage infiltration. A reduction in mechanical thresholds was observed from Week 8, coinciding with histopathological alterations in the tongue. At 16 and 20 wk, Iba‐1 immunoreactivity increased in the tongue and Sp5C, accompanied by morphological microglial changes, whereas nitrotyrosine and p‐p38 expression were elevated in the TG. These findings indicate a temporal association between peripheral tissue changes, nociceptive behavior, and glial/immune activation in both central and peripheral trigeminal structures, suggesting that neuroimmune interactions may contribute to the development of oral cancer pain.

## INTRODUCTION

Oral cancer pain reflects the unique anatomy and continuous functional activity of the oral cavity, arising from interactions between malignant neoplasms and peripheral nerve endings. The dense trigeminal innervation in this region contributes to intense pain at the primary tumor site, although central sensitization and referred pain may also occur through trigeminal pathways [1, 2, 3]. Orofacial pain typically worsens during oral function and is considered a key predictor for the progression from oral potentially malignant lesions (OPMLs) to oral cancer [2, 4, 5].

Previous studies have reported that pain is present in OPMLs and oral cancer [6, 7]. A study conducted by our group using an experimental model of oral carcinogenesis induced by 4‐nitroquinoline‐1‐oxide (4‐NQO) in rats demonstrated that nociceptive responses occur prior to cancer diagnosis and are exacerbated in animals with oral cancer. This increased nociceptive response is accompanied by increased c‐Fos expression in neurons of the trigeminal nociceptive pathway. Interestingly, c‐Fos expression was also detected in non‐neuronal cells, suggesting its possible involvement in oral cancer development [7].

An important feature of trigeminal nociceptive processing is the convergence of afferent inputs from different craniofacial territories onto second‐order neurons within the spinal trigeminal nucleus caudalis (Sp5C), the principal relay for orofacial pain [2, 3]. Because of this organization, persistent nociceptive input arising from one trigeminal branch may sensitize neurons that also receive signals from adjacent territories, leading to referred pain or secondary mechanical allodynia beyond the primary lesion site [3, 8]. Consistent with this concept, experimental models of tongue and gingival cancer have demonstrated reduced mechanical thresholds in the vibrissal pad following intraoral tumor induction, supporting the use of extraoral facial testing as a functional measure of trigeminal sensitization [9, 10].

Studies using the 4‐NQO model have documented a complex and heterogeneous immune infiltrate in the tongue, including neutrophils, myeloid cells (CD11b^+^), CD4^+^ and CD8^+^ T‐cells, regulatory T‐cells (CD4^+^CD25^+^FoxP3^+^), B‐cells, and myeloid‐derived suppressor cells, which change dynamically over the course of oral carcinogenesis [11]. Nevertheless, despite this complexity, macrophages appear quantitatively dominant in early‐stage lesions and may play a central role in shaping the tumor microenvironment [12], and they play a central role in tumor biology and neuroimmune interactions by releasing inflammatory mediators, modulating tissue remodeling, and influencing nociceptive signaling [13]. Although macrophage functions have been extensively studied in oncology, their specific temporal dynamics in oral carcinogenesis, as well as their interaction with neural elements along the trigeminal nociceptive pathway, remain poorly understood. Considering the central role of macrophages in regulating inflammation, tissue remodeling, and neuroimmune communication, characterizing their presence during oral carcinogenesis is essential to advance the understanding of pain‐related mechanisms in the 4‐NQO model.

Microglia and satellite glial cells (SGCs), both derived from neural crest cells, function similarly to macrophages by maintaining neural homeostasis and surveilling the microenvironment under physiological conditions. Following injury, these glial cells become activated, exhibiting hypertrophy, proliferation, morphological remodeling, including more arborescent structures, and upregulation of glial markers. They also release a variety of inflammatory mediators that contribute to neuroimmune interactions [2, 3, 14, 15].

Investigating the temporal changes in both peripheral immune cells and glial cells throughout the trigeminal nociceptive pathway is essential to address existing knowledge gaps in the mechanisms underlying oral carcinogenesis–related pain. This study aims to characterize the sequential activation of immune and glial cells in an experimental model of oral carcinogenesis, and to correlate these changes with nociceptive behavioral outcomes indicative of trigeminal sensitization.

## MATERIAL AND METHODS

### Animals

All experimental procedures were conducted in accordance with the National Institutes of Health (NIH) guidelines for the care and use of laboratory animals and complied with the ARRIVE guidelines. The study was approved by the Animal Ethics Committee of the Federal University of Ceará (protocol number 4861280219).

The sample size was calculated to detect a 20% difference between groups with a statistical power of 80%. Forty male Wistar rats (5–6‐wk old, 100–150 g) were obtained from the Central Animal Facility of the Federal University of Ceará. As this is the first study to investigate temporal associations between neuroimmune activation and nociceptive behavior during oral carcinogenesis, we used male rats to avoid potential confounding effects of the estrous cycle and hormonal fluctuations [16]. Furthermore, females generally display greater immune and glial reactivity than males, potentially influencing pain outcomes [17]. Restricting the study to males ensured greater consistency in the analysis of neuroinflammatory and nociceptive processes.

Animals were housed in polypropylene cages (three per cage) under controlled temperature and a 12‐h light/dark cycle, with irradiated wood shavings and environmental enrichment. Food and filtered water were provided ad libitum.

Rats were randomly assigned to the following groups using a computer‐generated sequence: Control (no exposure to 4‐NQO, *n* = 6), and 4‐NQO‐8W, 4‐NQO‐12W, 4‐NQO‐16W, and 4‐NQO‐20W (exposed to 4‐NQO for 8, 12, 16, or 20 wk, respectively). Animals in the carcinogenesis groups received 4‐NQO (0.3 mg/animal) applied to the dorsal surface of the tongue via topical dripping, twice daily (*n* = 8 per group). Mechanical allodynia was assessed throughout the experimental period using the von Frey test.

At the end of each exposure period, animals were anesthetized with ketamine (80 mg/kg) and xylazine (10 mg/kg), followed by transcardial perfusion with saline and 4% paraformaldehyde (PFA) for brain fixation. Tongues were harvested for immunohistochemical analysis.

In a second experimental protocol, the trigeminal ganglion (TG) was dissected at the level of the mandibular branch region, and the caudal subnucleus of the spinal trigeminal tract (Sp5C), site of the first synapse of the trigeminal nociceptive pathway was collected from 22 rats. These animals were divided into a Control group (no 4‐NQO exposure, *n* = 6) and experimental groups (4‐NQO‐16W and 4‐NQO‐20W; *n* = 8 per group) and were used for subsequent immunofluorescence analysis. These time points were selected a priori because they represented advanced stages of lesion progression and showed the lowest group mean mechanical thresholds in the initial behavioral analysis. Animals were not selected or excluded according to individual nociceptive responses. The Rat Grimace Scale was employed to assess pain‐related behaviors and to determine the need for humane euthanasia in animals exhibiting elevated distress scores.

### Mechanical allodynia

The animals were acclimated to the electronic von Frey apparatus (Insight) for 15 min/d over seven consecutive days in a sound‐attenuated behavioral testing room with reduced lighting. Mechanical allodynia was evaluated in the vibrissal pad region by a trained examiner blinded to the experimental groups (L.C.V.A.). Mechanical allodynia was evaluated in the vibrissal pad region by a trained examiner blinded to the experimental groups (L.C.V.A.). The vibrissal pad was selected as a surrogate site for the detection of secondary trigeminal mechanical allodynia. Persistent nociceptive input from tongue lesions (lingual nerve, V3 territory) may induce sensitization and convergence‐mediated hyperexcitability within the spinal trigeminal nucleus caudalis (Sp5C), resulting in reduced thresholds in adjacent facial receptive fields supplied by the maxillary division (V2), as previously reported in experimental oral cancer pain models [9, 10]. Direct mechanical stimulation of tongue lesions was intentionally avoided to prevent tissue injury, bleeding, and variability associated with progressive mucosal disruption during carcinogenesis.

A mechanical stimulus was applied, and the nociceptive threshold was defined as the force at which the animal exhibited a head‐withdrawal response. The stimulus was immediately removed upon the detection of this response [7]. To minimize the potential influence of induction‐related stress, the Von Frey test was performed 2 h after the second daily 4‐NQO administration.

### Histopathological analysis

Tongues were excised post‐euthanasia, fixed in 10% buffered formalin for 24 h, dehydrated, embedded in paraffin, and sectioned at 5 *µ*m. Sections were stained with hematoxylin and eosin (H&E) and examined by a pathologist blinded to the experimental groups, following World Health Organization criteria [18]. Epithelial dysplasia was scored as: 0 (no dysplasia); 1 (mild: with cytological and architectural changes restricted to basal/parabasal layers); 2 (moderate: abnormalities extending to basal/intermediate layers); 3 (severe: changes involving the full epithelial thickness) [18]. Oral squamous cell carcinoma (OSCC) was diagnosed by the presence of malignant epithelial cells arranged in nests and islands invading the connective tissue stroma (Figure 2).

The OSCCs were subsequently classified as single or multiple primary tumors. In addition, tumor morphology was categorized as papillary (exophytic lesions with an underlying invasive component), invasive (malignant epithelial cell infiltration to a depth of ≤2 mm), or deeply invasive (invasion exceeding 2 mm in depth) [19].

### Immunohistochemistry for Iba‐1 (tongue macrophages) and CD206

Histological sections were obtained from paraffin‐embedded tongue samples from animals subjected to oral carcinogenesis with 4‐NQO, euthanized at 0, 8, 12, 16, and 20 wk, to confirm the precise localization of tumor lesions. Tissue microarrays (TMAs) were constructed to enable simultaneous and standardized immunohistochemical analysis under identical staining conditions. Representative regions of the invasive front were carefully selected by an experienced pathologist on H&E‐stained slides and matched to the corresponding areas in the donor paraffin blocks. Selection criteria included regions exhibiting a dense infiltration of cancer cells with amoeboid morphology, loss of cell–cell adhesion, and remodeling of the extracellular matrix, features indicative of tumor progression at the invasive front. Two cylindrical tissue cores were extracted per animal using a 2‐mm punch and transferred to predefined positions in a recipient paraffin block to assemble the TMAs. One TMA slide was prepared for each antibody. The TMA blocks were sectioned at 5 *µ*m using a microtome, and the sections were mounted on silanized slides and stored at −20°C until use.

Immunohistochemistry for Iba‐1 and CD206 was performed on paraffin‐embedded sections using the streptavidin–biotin–peroxidase method. Iba‐1 was used to label macrophages, and CD206 was employed to identify M2 macrophage subtypes at the invasive front of tongue lesions. Following deparaffinization and antigen retrieval, sections were incubated overnight at 4°C with either anti–Iba‐1 (1:200; Fujifilm Wako Chemicals) or anti‐CD206 (1:5000; Sigma‐Aldrich), diluted in DAKO antibody diluent. Subsequently, sections were incubated for 30 min with the polymer‐based secondary reagent (Invision Flex HRP, DAKO).

Immunoreactivity was visualized using a diaminobenzidine (DAB)‐H_2_O_2_ chromogenic solution, resulting in brown cytoplasmic staining in positive macrophages. A calibrated examiner blinded to the experimental groups randomly selected five high‐power fields (400× magnification) per sample for cell counting using imagej software (K.M.A.P.). Cells with cytoplasmic or nuclear positivity for Iba‐1 and/or CD206 were quantified, and mean values per sample were used for statistical analysis. Lymph node and liver tissues served as positive controls for Iba‐1 and CD206, respectively.

### Immunofluorescence analysis for nitrotyrosine, phospho‐p38 MAP kinase, synthetase glutamine, and Iba‐1

Following the completion of the first experimental protocol, 22 animals were randomized into a Control group (*n* = 6) and two predefined experimental groups corresponding to 16 and 20 wk of 4‐NQO exposure (*n* = 8 each). These time points were selected a priori for subsequent TG and Sp5C analyses because, in the behavioral assessment, they collectively exhibited the most pronounced reduction in nociceptive thresholds. Importantly, animals were not selected or excluded based on individual nociceptive responses. All animals that completed the respective exposure periods were included.

After euthanasia, the TG and Sp5C tissues were dissected, fixed in 4% PFA for 2 h, and cryoprotected in 30% sucrose solution for 48 h. TG analyses were focused on the mandibular division (V3) region, corresponding to the principal sensory territory of the lingual nerve. Brainstem analyses were performed in the spinal trigeminal nucleus caudalis (Sp5C), the first central relay of trigeminal nociceptive afferents.

Subsequently, the tissues were embedded in Tissue‐Tek OCT compound and stored at −80°C. For immunohistochemical analyses of nitrotyrosine, p‐p38, GC, three nonconsecutive sections per animal from the mandibular branch region of the TG were analyzed. Iba‐1 analyses in the Sp5C were performed using three nonconsecutive sections per animal, following the same sampling approach.

For the immunofluorescence assay, TG and Sp5C samples were sectioned at 10 *µ*m thickness using a cryostat (Leica CM1100). Antigen retrieval was performed in sodium citrate buffer, followed by permeabilization with 0.1% Triton X‐100 (Sigma‐Aldrich) for 10 min. To reduce non‐specific binding, sections were incubated in 0.3 M glycine and 5% bovine serum albumin (Sigma‐Aldrich) for 30 min.

The TG samples were incubated overnight (2–8°C) with primary antibodies in rabbit anti‐phospho‐p38 MAP kinase (p‐p38) (1:400; Cell Signaling Technology), or anti‐nitrotyrosine (1: 200; Millipore). Subsequently, incubation was performed with the secondary antibody, goat anti‐rabbit IgG Alexa Fluor 568 (1:400; Invitrogen, Life Technologies, Thermo Fisher Scientific). For the labeling of SGCs, the primary antibody used was mouse anti‐glutamine synthetase (Millipore) (dilution of 1:200), followed by the secondary antibody, goat anti‐mouse IgG Alexa Fluor 633 (1:400; Invitrogen, Life Technologies, Thermo Fisher Scientific). For the labeling of neuronal cell bodies, the TG samples were also incubated with the mouse anti‐NeuN antibody conjugated with Alexa Fluor 488 (1:100; Merck Millipore).

The samples from the Sp5C region were incubated overnight with the primary antibody rabbit anti‐Iba‐1 (1:200; Cell Signaling Technology), followed by incubation with the secondary antibody goat anti‐rabbit IgG Alexa Fluor 568 (1:400; Invitrogen, Life Technologies, Thermo Fisher Scientific). To label the cell nuclei in both TG and Sp5C, samples were incubated with DAPI (4*µ*l in 200ml of PBS; Invitrogen, Life Technologies, Thermo Fisher Scientific). The slides were mounted using Prolong Gold Antifade Mountant (Invitrogen, Life Technologies, Thermo Fisher Scientific). Photomicrographs were acquired using a laser scanning confocal microscope (Zeiss LSM 710, Carl Zeiss).

The quantification of the fluorescent area was performed using an image analysis software (Fiji ImageJ, version 1.51k; National Institutes of Health). Initially, an experienced examiner (A.F.P.), blinded to the experimental groups, standardized the lower and upper limits to set the selected and unselected pixels by the color threshold. Then, the fluorescent pixels were differentiated from non‐fluorescent by the higher color saturation related to the observed fluorescence (red or green). For nitrotyrosine and p‐p38, the results were expressed as the percentage of the positive fluorescent area normalized to NeuN immunofluorescence, which was considered 100. For Iba‐1, the quantification was expressed as the percentage of the positively labeled area relative to the total analyzed area. For SGCs, the total immunostained area was divided by the number of NeuN‐positive neurons in the photomicrographs, yielding the mean immunoreactive area per neuron [20].

### Microglia and SGC morphological analysis

The overall immunostained area was divided by the total number of neuron spots in the photomicrographs to determine the average area of SGCs surrounding neurons [20]. For microglial morphology assessment, Iba‐1‐stained photomicrographs were captured with maximum pinhole aperture then converted to 8‐bit grayscale. Images were binarized using ImageJ software (Bethesda; RRID) and processed with the Skeletonize plugin. The mean number of branch endpoints and total branch length per cell were calculated [21].

### Statistical analysis

The Shapiro–Wilk test confirmed a parametric data distribution. Longitudinal behavioral data were analyzed using two‐way repeated‐measures anova followed by Bonferroni post hoc tests. Comparisons among multiple independent groups were performed using one‐way anova followed by Tukey or Dunnett's post hoc tests, as appropriate. Comparisons between two groups were analyzed using Student's *t*‐test. Results were expressed as mean ± standard error of the mean (SEM), with significance set at *p* < 0.05. Analyses were conducted using GraphPad Prism 8.0.

## RESULTS

### 4‐NQO exposure induces mechanical allodynia

**FIGURE 1:**
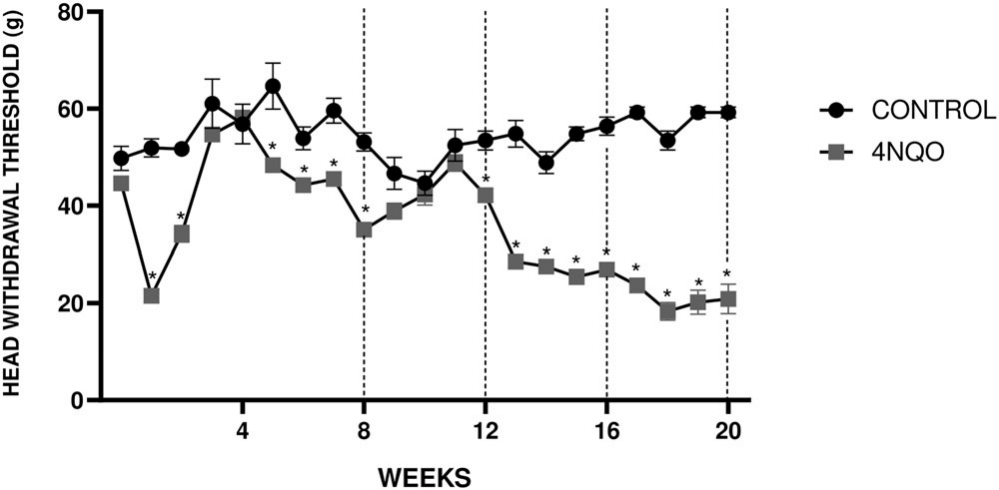
Nociceptive response during 4‐NQO‐induced oral carcinogenesis in rats. Development of mechanical allodynia in rats subjected to 4‐NQO‐induced oral carcinogenesis. Data are shown as individual points with mean ± SEM (Control, *n* = 6; 4‐NQO groups, *n* = 8 per group). Statistical comparisons were performed using two‐way repeated‐measures anova followed by Bonferroni post hoc tests to compare 4‐NQO groups with Control at each time point. *p* < 0.05 versus Control. 4‐NQO = 4‐nitroquinoline 1‐oxide; Control = animals not receiving 4‐NQO; 4‐NQO‐8W = 4‐NQO applied to the tongue (2×/d) for 8 wk; 4‐NQO‐12W = 4‐NQO (2×/d) for 12 wk; 4‐NQO‐16W = 4‐NQO (2×/d) for 16 wk; 4‐NQO‐20W = 4‐NQO (2×/d) for 20 wk.

The head withdrawal threshold was assessed throughout the experiment by comparing animals exposed to the carcinogen for 4, 8, 12, 16, or 20 wk with the Control group. A significant reduction in threshold was observed in all 4‐NQO groups from Week 8 onward (*p* = 0.02), except at Week 12, which did not differ from Control. Both the 16‐ and 20‐wk exposure groups exhibited markedly reduced nociceptive thresholds compared with the Control group (*p* < 0.0001) (Figure 1). Additionally, no statistically significant differences were observed between animals showing OPMLs and those with OSCCs (Figure S1). Grimace scale analysis indicated that no animals required removal due to signs of excessive suffering (Table S1).

### Histopathological alterations induced by 4‐NQO in the rat tongue

**FIGURE 2:**
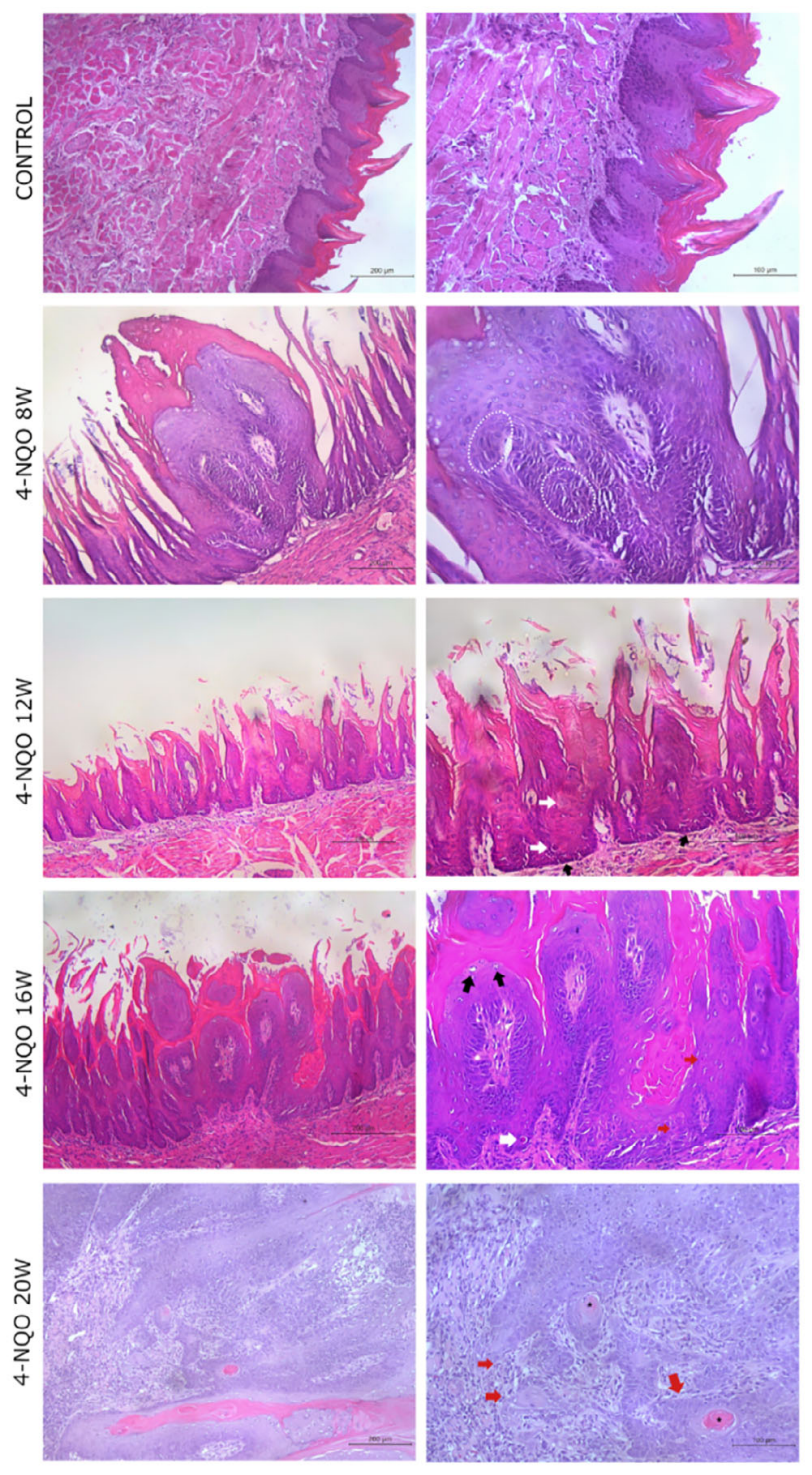
Representative tongue lesions during 4‐NQO‐induced carcinogenesis in rats. (Control = 6 animals; 4‐NQO groups = 8 animals/group). Control: normal epithelium; 4‐NQO‐8W: mild dysplasia; 4‐NQO‐12W: mild dysplasia; 4‐NQO‐16W: moderate dysplasia; 4‐NQO‐20W: oral squamous cell carcinoma. Left column: 100× magnification; right column: 200× magnification. Hematoxylin and eosin staining. 4‐NQO = 4‐nitroquinoline 1‐oxide; Control = animals not receiving 4‐NQO; 4‐NQO‐8W = 4‐NQO applied to the tongue (2×/d) for 8 wk; 4‐NQO‐12W = 4‐NQO (2×/d) for 12 wk; 4‐NQO‐16W = 4‐NQO (2×/d) for 16 wk; 4‐NQO‐20W = 4‐NQO (2×/d) for 20 wk. In 4‐NQO‐8W: white circular area = alterations restricted to the basal–parabasal layer epithelium layer. In 4‐NQO‐12W: black arrow = drop‐shaped epithelial ridges; white arrow = cellular pleomorphism. In 4‐NQO‐16W: black arrow = cellular pleomorphism; white arrow = dyskeratosis; red arrow = atypical mitosis; In 4‐NQO‐20W: black asterisk = keratin pearl; red arrow = sheets and nests of neoplastic cells.

No histopathological alterations were detected in the Control group, in which all samples displayed normal epithelium. After 8 wk of 4‐NQO exposure, epithelial changes emerged, with most samples showing mild dysplasia (62.5%) and the remainder presenting moderate dysplasia (37.5%). At 12 wk, 75% of the lesions were classified as mild dysplasia associated with hyperkeratosis, whereas 25% of the tongues exhibited isolated hyperkeratosis (Figure 2, Table S2). From 16 wk onward, a clear progression in lesion severity was evident, with the appearance of severe dysplasia (25%) and the first cases of OSCC (12.5%), characterized by a single deeply invasive lesion. By 20 wk, the disease burden had shifted toward advanced stages, with OSCC affecting half of the samples and the remainder showing varying degrees of dysplasia (Figure 2). OSCC at 20 wk displayed papillary exophytic lesions (100%), with multiple primary tumors observed in 60% of cases (Table S3).

### Increased Iba‐1^+^ tongue macrophages and absence of CD206 expression in the tongues of rats subjected to 4‐NQO‐induced oral carcinogenesis

**FIGURE 3:**
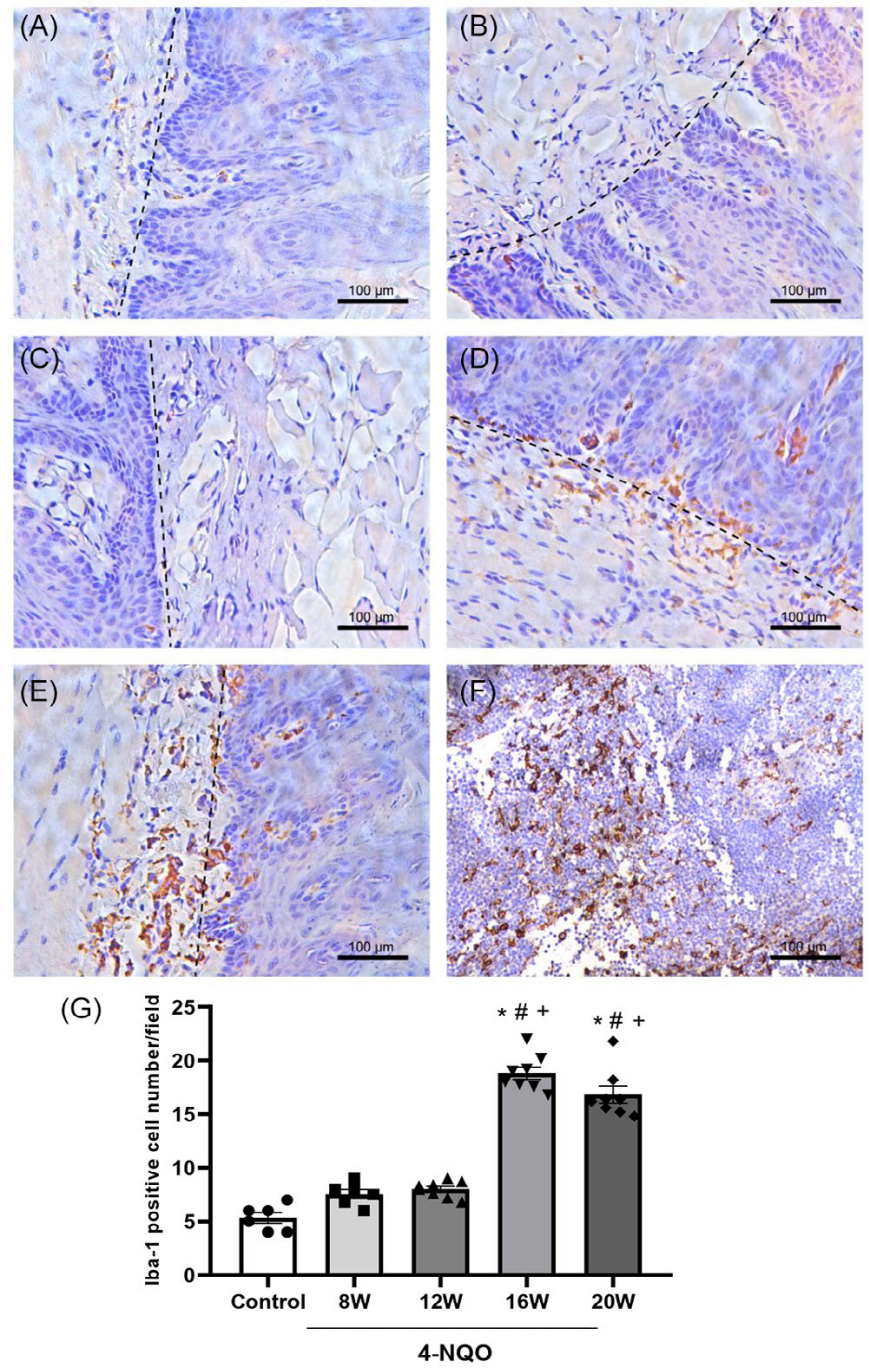
Photomicrographs (A–E) and quantitative analysis (G) of Iba^+^ tongue macrophages immunoexpression in rats subjected to 4‐NQO‐induced oral carcinogenesis. Data are shown as individual points with mean ± SEM (Control = 6 animals; 4‐NQO groups = 8 animals/group). Statistical comparisons were performed using one‐way anova followed by Tukey post hoc test when appropriate. ^*^ *p *< 0.0001 versus Control. ^#^ *p *< 0.005 versus 4‐NQO‐8W. ^+^ *p *< 0.005 versus 4‐NQO‐12W. (A) Control = animals not receiving 4‐NQO. (B) 8W: 4‐NQO applied to the tongue (2×/d) for 8 wk. (C) 12W: 4‐NQO (2×/d) for 12 wk. (D) 16W: 4‐NQO (2×/d) for 16 wk. (E) 20W: 4‐NQO (2×/d) for 20 wk. (F) Positive control: lymph node. Yellow dashed line = boundary between the epithelial and connective tissues.

There was a significant increase in Iba‐1^+^ tongue macrophages in the 4‐NQO‐16W and 4‐NQO‐20W groups compared to the Control group (*p* < 0.0001). Significant differences were also found when comparing the 4‐NQO‐16W (*p* < 0.0001) and 4‐NQO‐20W (*p* = 0.0004) groups with the 4‐NQO‐8W group (Figure 3A–E). Additionally, the 4‐NQO‐16W (*p* < 0.0001) and 4‐NQO‐20W (*p* = 0.0015) groups showed increased Iba‐1 expression compared to the 12‐wk exposure group (Figure 3C–E). However, no statistical difference was noted between the 4‐NQO‐16W and 4‐NQO‐20W groups (Figure 3G). Figure 3F represents the positive control used for Iba‐1 detection. No CD‐206 immunoexpression was detected in any tongue regions across all groups (Figure S2).

### Increased nitrotyrosine, p‐p38, and glutamine synthetase in the trigeminal ganglion, and elevated Iba‐1 expression in the Sp5C region

Considering the results on nociceptive behavior and immunohistochemical analysis of Iba1^+^ tongue macrophages, the immunofluorescence assay of the nociceptive trigeminal pathway was performed using a single control group and the 4‐NQO groups that exhibited increased nociceptive responses and Iba‐1 immunostaining (4‐NQO‐16W and 4‐NQO‐20W). Control animals were timepoint‐matched to the corresponding 4‐NQO groups, and no significant differences were observed between controls at 16 and 20 wk.

**FIGURE 4:**
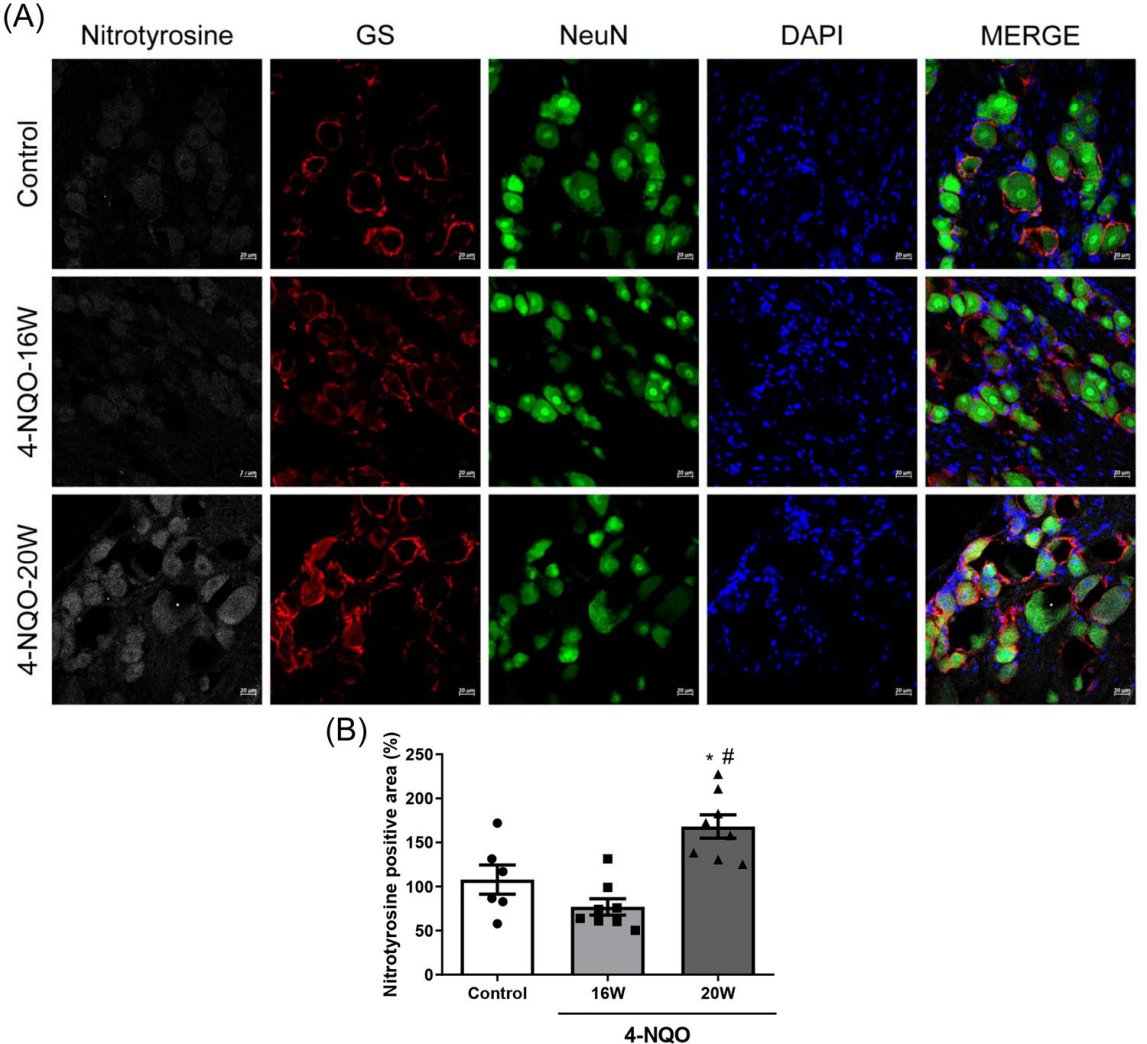
Photomicrographs (A) and quantification of the fluorescent area (B) of nitrotyrosine expression in the trigeminal ganglion of rats subjected to 4‐NQO‐induced oral carcinogenesis. Scale: 20 *µ*m. Data are shown as individual points with mean ± SEM (Control = 6 animals; 4‐NQO groups = 8 animals/group). Statistical comparisons were performed using one‐way anova followed by Tukey post hoc test when appropriate. ^*^ *p* < 0.05 versus Control. ^#^ *p* < 0.005 versus 4‐NQO‐16W. 4‐NQO = 4‐nitroquinoline 1‐oxide; Control = animals not receiving 4‐NQO; 16W = 4‐NQO applied to the tongue (2×/d) for 16 wk; 20W = 4‐NQO applied to the tongue (2×/d) for 20 wk. White: nitrotyrosine; red: glutamine synthetase; green: NeuN (neuronal marker); blue: DAPI (nuclei marker).

**FIGURE 5:**
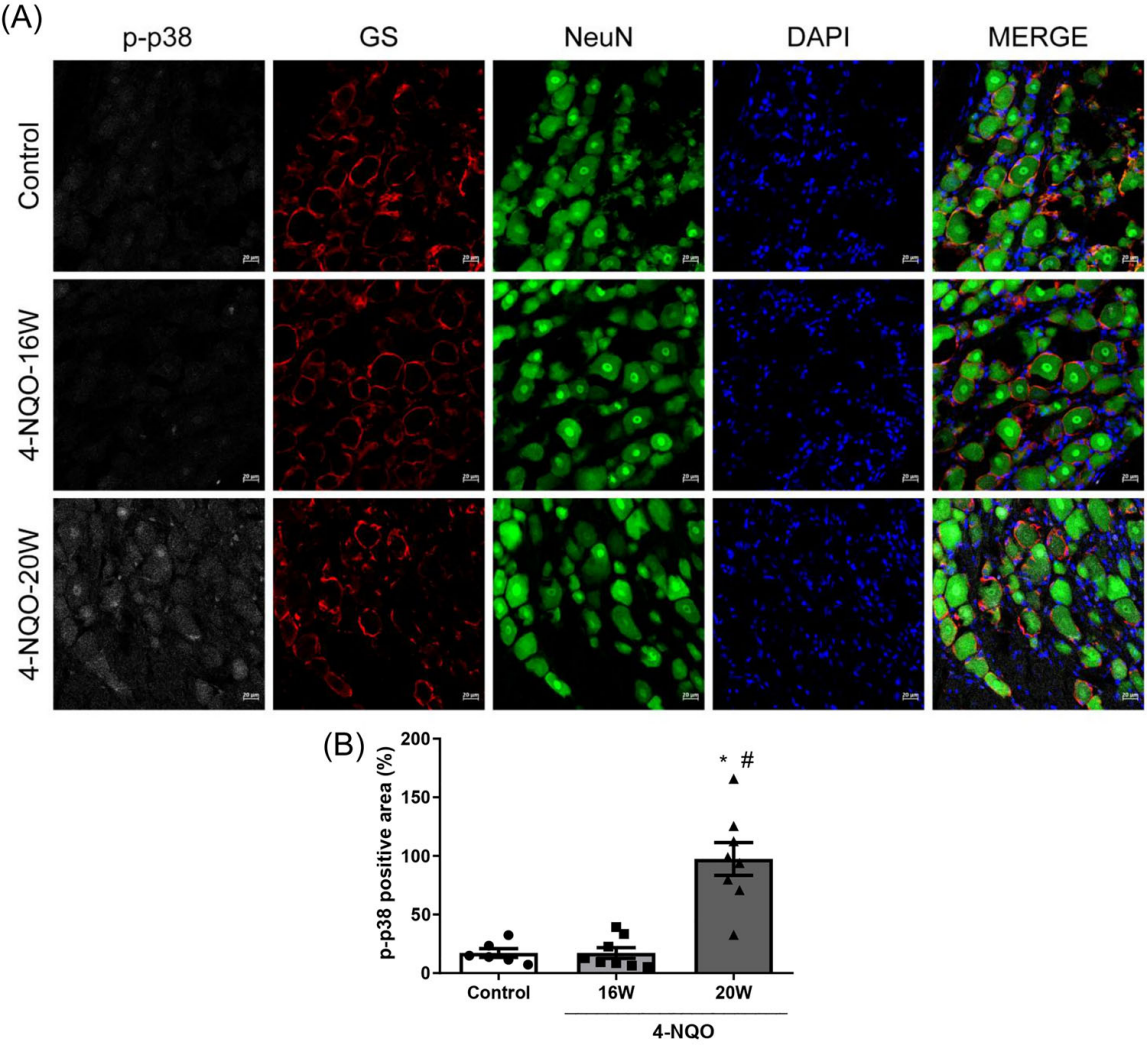
Photomicrographs (A) and quantification of the fluorescent area (B) of p‐p38 expression in the trigeminal ganglion of rats subjected to 4‐NQO‐induced oral carcinogenesis. Scale: 20 *µ*m. Data are shown as individual points with mean ± SEM (Control = 6 animals; 4‐NQO groups = 8 animals/group). Statistical comparisons were performed using one‐way anova followed by Tukey post hoc test when appropriate ^*^ *p* < 0.005 versus Control. ^#^ *p* < 0.005 versus 4‐NQO‐16W. 4‐NQO = 4‐nitroquinoline 1‐oxide; Control = animals not receiving 4‐NQO; 16W = 4‐NQO applied to the tongue (2×/d) for 16 wk; 20W = 4‐NQO applied to the tongue (2×/d) for 20 wk. White: p‐p38; red: glutamine synthetase; green: NeuN (neuronal marker); blue: DAPI (nuclei marker).

An increased nitrotyrosine immunolabeling was observed in the neuronal cytoplasm of the TG in animals exposed to 4‐NQO for 20 wk compared with both the Control group (*p* = 0.049) and the 4‐NQO‐16W group (*p* = 0.005) (Figure 4A,B). In addition, p‐p38 immunostaining was significantly increased at 20 wk in both SGCs and neuronal nuclei compared with all other groups (*p* = 0.0011 and *p* = 0.0016, respectively) (Figure 5A,B).

**FIGURE 6:**
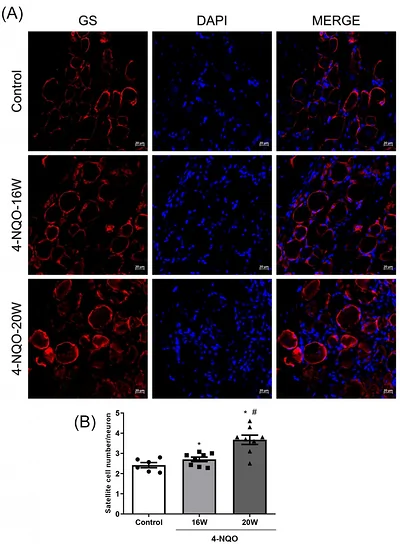
Photomicrographs of immunofluorescence for glutamine synthetase (A) and quantification of satellite cells (B) in the trigeminal ganglion. Scale: 20 *µ*m. Data are shown as individual points with mean ± SEM (Control = 6 animals; 4‐NQO groups = 8 animals/group). Statistical comparisons were performed using one‐way anova followed by Tukey post hoc test when appropriate. ^*^ *p* < 0.05 versus Control. ^#^ *p* < 0.0001 versus 4‐NQO‐16W. 4‐NQO = 4‐nitroquinoline 1‐oxide; Control = animals not receiving 4‐NQO; 16W = = 4‐NQO applied to the tongue (2×/d) for 16 wk; 20W = 4‐NQO applied to the tongue (2×/d) for 20 wk. Red: glutamine synthetase; blue: DAPI (nuclei marker).

**FIGURE 7:**
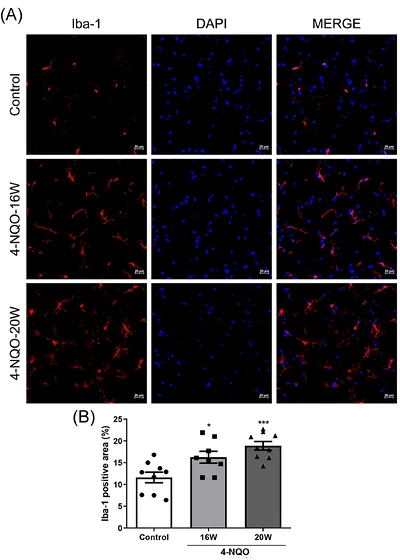
Photomicrographs (A) and quantification of the fluorescent area (B) of Iba‐1 expression in the Sp5C of rats subjected to 4‐NQO‐induced oral carcinogenesis. Scale: 20 *µ*m. Data are shown as individual points with mean ± SEM (Control = 6 animals; 4‐NQO groups = 8 animals/group). Statistical comparisons were performed using one‐way anova followed by Tukey post hoc test when appropriate. ^*^ *p* < 0.05 versus Control. ^***^ *p* < 0.001 versus Control. 4‐NQO = 4‐nitroquinoline 1‐oxide; Control = animals not receiving 4‐NQO; 16W = 4‐NQO applied to the tongue (2×/d) for 16 wk; 20W = 4‐NQO applied to the tongue (2×/d) for 20 wk. Red: Iba‐1; blue: DAPI (nuclei marker).

Regarding the number of glial cells, there was an increase in SGCs in the TG at the 16th (*p* = 0.045) and 20th wk (*p* < 0.0001) of carcinogen exposure compared to the Control. The 4‐NQO‐20W group showed a higher number of SGCs compared to the 4‐NQO‐16W group (*p* = 0.0079) (Figure 6A,B). In the Sp5C region, both the 4‐NQO‐16W (*p* = 0.0128) and 4‐NQO‐20W (*p* = 0.0009) groups exhibited increased expression of Iba‐1 compared to the Control group. However, there was no significant difference in the count of microglial cells between the 4‐NQO groups (Figure 7A,B).

### Microglia morphological analysis

**FIGURE 8:**
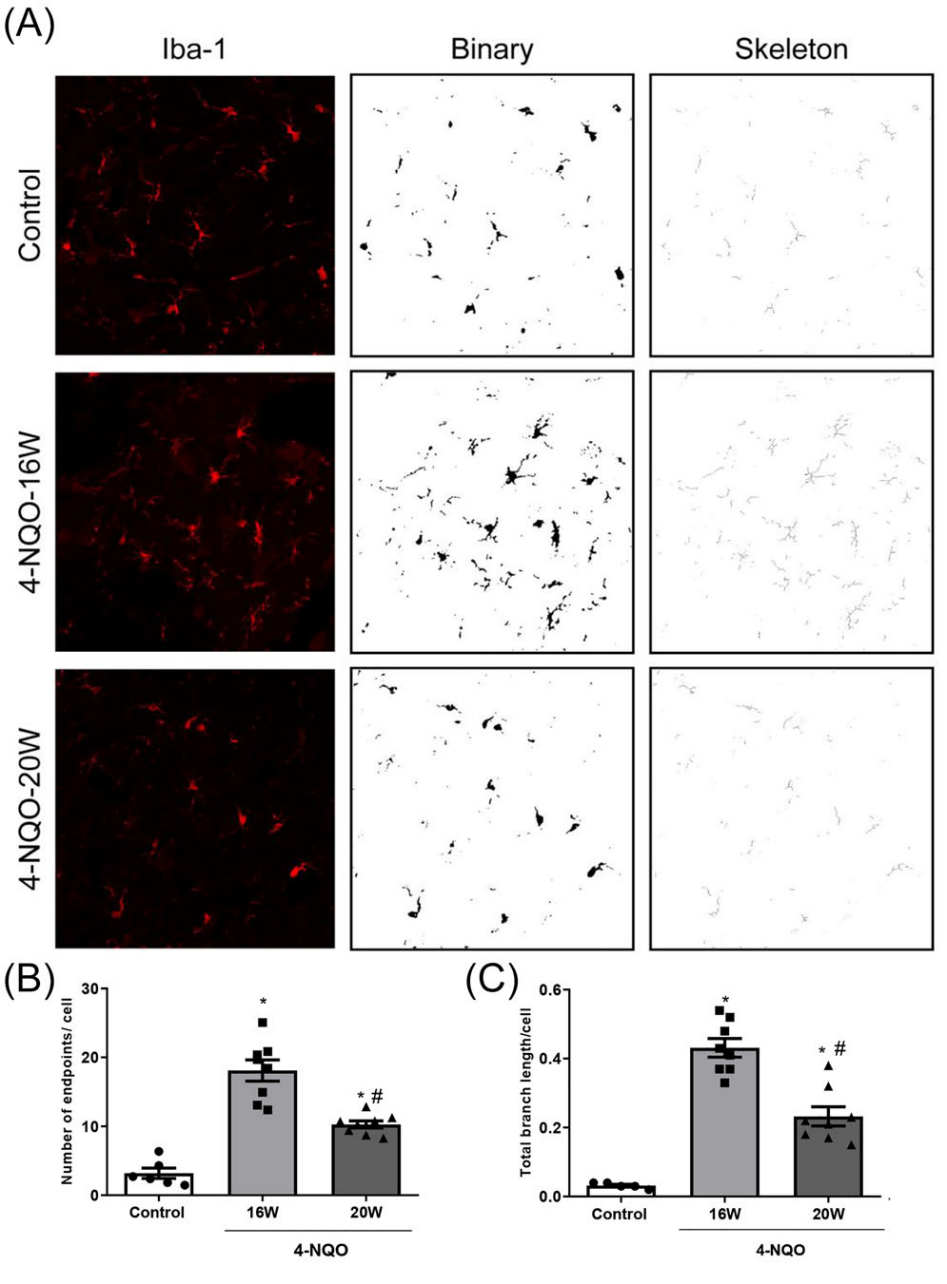
(A) Photomicrographs of microglial morphology in the Sp5C region, presented as binarized and skeletonized images (200× magnification). Red: Iba‐1 staining. (B) Quantification of microglial endpoints. (C) Quantification of total microglial branch length in the Sp5C region of rats subjected to 4‐NQO‐induced oral carcinogenesis. Data are shown as individual points with mean ± SEM (Control = 6 animals; 4‐NQO groups = 8 animals/group). Statistical comparisons were performed using one‐way anova followed by Tukey post hoc test when appropriate ^*^ *p* < 0.05 versus Control. ^#^ *p* < 0.05 versus 4‐NQO‐16W. 4‐NQO = 4‐nitroquinoline 1‐oxide; Control = animals not receiving 4‐NQO; 16W = 4‐NQO applied to the tongue (2×/d) for 16 wk; 20W = 4‐NQO applied to the tongue (2×/d) for 20 wk.

A significant increase in both the length and number of microglial branches in the Sp5C region was observed in the 4‐NQO‐16W group compared to the Control group (branch length: *p* < 0.0001; branch number: *p* < 0.0001) (Figure 8A,B). The 4‐NQO‐20W group also exhibited a significant increase in branch length (*p* = 0.0015) and number (*p* = 0.0208) relative to controls. However, when compared to the 4‐NQO‐16W group, the 4‐NQO‐20W group showed a significant reduction in both parameters (length: *p* = 0.0046; number: *p* = 0.0119) (Figure 8A–C).

## DISCUSSION

This study addresses a critical gap in the literature regarding the temporal dynamics of neuroimmune activation during oral carcinogenesis. Most previous work on oral cancer–related pain has emphasized neuronal mechanisms, with a limited exploration of immune and glial contributions to the trigeminal nociceptive pathway. By integrating these non‐neuronal components, we established a temporal sequence linking carcinogen exposure to nociceptive alterations in rats, revealing an early activation of SGCs and microglia/macrophages in the Sp5C region, followed by macrophage recruitment and activation in the tongue. These time‐dependent cellular events provide insights into the pathophysiology of oral cancer pain and may inform the design of targeted therapeutic strategies.

Macrophages are recognized as important contributors to oral carcinogenesis, particularly through their ability to sustain local inflammation, release cytokines and chemokines, and modulate tissue remodeling [22, 23]. Continuous 4‐NQO exposure in our model led to increased Iba‐1 immunolabeling in the tongue, consistent with enhanced macrophage infiltration and activation, which likely contributes to the nociceptive alterations observed throughout carcinogenesis. Future studies employing approaches to modulate peripheral macrophage activity will be critical to elucidate their specific role in oral cancer–associated pain.

Tumor progression induces a proinflammatory environment combined with nerve compression, perineural invasion, ischemia, and tissue acidosis, all contributing to the sensitization of oral primary afferent fibers [24]. Notably, mechanical hypersensitivity observed during the early weeks of 4‐NQO exposure likely reflects transient local irritation by the carcinogen, as previously reported in our group's study [7]. As exposure continued, compensatory changes in the tongue epithelium, including hyperkeratosis, acted as a physical barrier and partially restored nociceptive thresholds around 12 wk. At later stages (16–20 wk), corresponding to advanced dysplastic and malignant changes, mechanical hypersensitivity re‐emerged and was accompanied by increased Iba‐1 expression in the tongue, indicating macrophage infiltration, as well as enhanced activation of glial cells in trigeminal structures. These findings suggest that early hypersensitivity reflects inflammatory mechanisms triggered by carcinogen exposure, whereas sustained or renewed nociceptive alterations at later stages are associated with progressive neuroimmune activation during oral carcinogenesis.

Mechanical sensitivity in the vibrissal pad was selected based on our previous characterization of this 4‐NQO model, in which extraoral facial hypersensitivity emerged during oral carcinogenesis and was accompanied by increased c‐Fos expression in trigeminal nociceptive structures [7]. The present study extends those findings by investigating whether this previously established behavioral phenotype is temporally associated with activation of peripheral and central non‐neuronal cells along the trigeminal pathway. Such extraoral hypersensitivity is consistent with trigeminal convergence mechanisms, whereby persistent lingual afferent input (V3 territory) may sensitize second‐order neurons in the spinal trigeminal nucleus caudalis (Sp5C), contributing to referred pain and reduced thresholds in adjacent facial territories [25, 26].

Alves *et al.* [7] demonstrated that the enhanced activation of trigeminal nociceptive pathways became evident at later stages of carcinogenesis (16 wk), concomitant with increased c‐Fos expression in neurons of the TG and the spinal trigeminal nucleus caudalis (Sp5C). In addition to neuronal labeling, c‐Fos immunoreactivity was also detected in non‐neuronal cells, indicating an early involvement of glial and immune components during disease progression [7]. This temporal pattern suggests that neuronal activation occurs in parallel with, and may precede, neuroimmune responses within the trigeminal pathway. These observations provided the rationale for the present study, which systematically investigates the sequential activation of non‐neuronal cell populations and their association with nociceptive alterations throughout oral carcinogenesis.

Control animals showed minor fluctuations in nociceptive thresholds over 20 wk, without statistical significance, a pattern consistent with transient stress responses previously reported in rodents [27, 28].

SGCs in the TG form functional units with neurons, regulating ionic homeostasis, neurotransmitter recycling, and amplifying nociceptive signaling [29]. Under basal conditions, SGCs express low levels of GFAP and glutamine synthetase, but after activation these markers increase, along with the number of SGCs per neuron [23, 28]. Activated SGCs release IL‐1β and TNF‐α, propagate calcium waves via gap junctions, and indirectly sensitize adjacent neurons within the TG. Although this local glial–neuronal interaction amplifies nociceptive signaling, the emergence of reduced thresholds at the vibrissal pad, despite lesions being confined to the tongue, is primarily driven by the subsequent central sensitization and convergence of these signals onto second‐order neurons within the Sp5C [7, 9].

Accordingly, the von Frey assessment in the vibrissal pad should be interpreted as a functional readout of referred trigeminal hypersensitivity rather than as a direct measure of nociception at the lesion site. This is consistent with the somatotopic organization of trigeminal‐mediated pain, in which neurons in the caudal subnucleus of the spinal trigeminal nucleus (Sp5C) represent concentric “onion‐skin” layers across the orofacial region, with overlapping representations of the tongue and perioral areas. Thus, mechanical allodynia observed in the vibrissal pad likely captures the referred component of oral lesion–related nociception. Sustained inflammation further enhances SGC activation through p38 MAPK phosphorylation, contributing to persistent and neuropathic‐like pain [9, 14, 30].

Similarly, microglia in the Sp5C exhibited increased Iba‐1 expression after 16–20 wk of 4‐NQO exposure, paralleling heightened nociceptive responses. Morphometric analysis revealed hyper‐ramified microglia at 16 wk and reactive morphology at 20 wk, with enlarged soma and reduced branching. These findings suggest progressive microglial remodeling in response to chronic peripheral injury. Microglial activation is central to trigeminal hyperexcitability, as these cells release proinflammatory mediators and undergo cytoskeletal reorganization that sustains central sensitization [10, 31, 32, 33].

The increase in nitrotyrosine expression in the 4‐NQO‐20W group indicates elevated nitrosative stress, potentially driving p‐p38 expression in TG neurons and SGCs. Nitric oxide, produced by macrophages, endothelial cells, and neurons, contributes to DNA damage, angiogenesis, and immune suppression [34, 35, 36]. The temporal overlap among nitrosative stress, p‐p38 signaling, and glial activation aligns with the progressive nociceptive behavior observed in this model.

Our results reveal coordinated changes in peripheral tissues, nociceptive behavior, and central glial/immune activation, supporting the notion that central nociceptive pathways are engaged early in oral carcinogenesis, potentially contributing to heightened pain sensitivity. Although causality cannot be inferred from our study, our data indicate that immune and glial cells are temporally associated with the development of pain during oral carcinogenesis, suggesting a potential contributory role. The study was designed to investigate temporal associations among tongue pathology, nociceptive behavior, and neuroimmune activation rather than to directly interrogate neuronal circuitry. Future studies using electrophysiology, neuronal tracing, functional markers, live imaging, transcriptomics, or cell‐specific manipulations will be essential to clarify these interactions and their mechanistic relevance to oral cancer–associated pain.

In conclusion, pain during oral carcinogenesis is associated with complex, early neuroimmune interactions involving both peripheral and central components of the trigeminal system. The engagement of glial and immune cells prior to tumor malignancy suggests that neuroimmune mechanisms may contribute to the development of cancer‐related pain. These findings highlight potential targets for early intervention, moving beyond symptom management toward modulation of pain‐related pathways during oral carcinogenesis.

## AUTHOR CONTRIBUTIONS

**Conceptualization**: Delane Viana Gondim and Karuza Maria Alves Pereira. **Methodology**: Delane Viana Gondim, Roberto César Pereira Lima Júnior, and Mariana Lima Vale. **Investigation**: Joana Maria dos Santos Alves, Luiza Clertiani Vieira Alves, Vitória Tavares Bessa, Anamaria Falcão Pereira, and Mariana Lima Vale. **Formal analysis**: Joana Maria dos Santos Alves, Luiza Clertiani Vieira Alves, and Karuza Maria Alves Pereira. **Data curation**: Roberto César Pereira Lima Júnior. **Project administration**: Delane Viana Gondim. **Supervision**: Delane Viana Gondim and Karuza Maria Alves Pereira. **Writing—draft**: Joana Maria dos Santos Alves and Luiza Clertiani Vieira Alves. **Writing—review and editing**: Joana Maria dos Santos Alves and Delane Viana Gondim.

## CONFLICT OF INTEREST STATEMENT

The authors declare no conflicts of interest.

## FUNDING INFORMATION

This research did not receive any specific grant from funding agencies in the public, commercial, or not‐for‐profit sectors.

## Supporting information



Supporting Information

Supporting Information

Supporting Information

Supporting Information

Supporting Information

## Data Availability

Data will be made available on request.

## References

[1] Viet CT , Schmidt BL . Biologic mechanisms of oral cancer pain and implications for clinical therapy. J Dent Res. 2012;91:447–53. 10.1177/0022034511424156 PMC3327727 21972258

[2] Ye Y , Salvo E , Romero‐Reyes M , Akerman S , Shimizu E , Kobayashi Y , et al. Glia and orofacial pain: progress and future directions. Int J Mol Sci. 2021;22:5345. 10.3390/ijms22105345 PMC8160907 34069553

[3] Shinoda M , Hayashi Y , Kubo A , Iwata K . Pathophysiological mechanisms of persistent orofacial pain. J Oral Sci. 2020;62:131–5. 10.2334/josnusd.19-0373 32132329

[4] Romero‐Reyes M , Salvemini MD . Cancer and orofacial pain. Med Oral Patol Oral Cir Bucal. 2016;21:e665–71. 10.4317/medoral.21515 PMC5116107 27694791

[5] Salvo E , Campana WM , Scheff NN , Nguyen TH , Jeong SH , Wall I , et al. Peripheral nerve injury and sensitization underlie pain associated with oral cancer perineural invasion. Pain. 2020;161:2592–602. 10.1097/j.pain.0000000000001986 PMC7572698 32658150

[6] Lam DK , Schmidt BL . Orofacial pain onset predicts transition to head and neck cancer. Pain. 2011;152:1206–9. 10.1016/j.pain.2011.02.009 PMC3099418 21388740

[7] Alves JMS , Viana KF , Pereira AF , Lima Júnior RCP , Vale ML , Pereira KMA , et al. Oral carcinogenesis triggers a nociceptive behavior and c‐Fos expression in rats’ trigeminal pathway. Oral Dis. 2023;29:1531–41. 10.1111/odi.14176 35244314

[8] Shinoda M , Hitomi S , Iwata K , Hayashi Y . Plastic changes in nociceptive pathways contributing to persistent orofacial pain. J Oral Biosci. 2022;64:263–70. 10.1016/j.job.2022.07.001 35840073

[9] Hironaka K , Ozaki N , Hattori H , Nagamine K , Nakashima H , Ueda M , et al. Involvement of glial activation in trigeminal ganglion in a rat model of lower gingival cancer pain. Nagoya J Med Sci. 2014;76:323–32. PMID: 25741041 PMC4345692 25741041

[10] Tamagawa T , Shinoda M , Honda K , Furukawa A , Kaji K , Nagashima H , et al. Involvement of microglial P2Y12 signaling in tongue cancer pain. J Dent Res. 2016;95:1176–82. 10.1177/0022034516647713 27151915

[11] Yamaguchi H , Hiroi M , Mori K , Ushio R , Matsumoto A , Yamamoto N , et al. Simultaneous expression of Th1‐ and Treg‐associated chemokine genes and CD4^+^, CD8^+^, and Foxp3^+^ cells in the premalignant lesions of 4NQO‐induced mouse tongue tumorigenesis. Cancers (Basel). 2021;13:1835. 10.3390/cancers13081835 PMC8069711 33921389

[12] Bouaoud J , Foy JP , Tortereau A , Michon L , Lavergne V , Gadot N , et al. Early changes in the immune microenvironment of oral potentially malignant disorders reveal an unexpected association of M2 macrophages with oral cancer free survival. Oncoimmunology. 2021;10:1944554. 10.1080/2162402X.2021.1944554 PMC8238000 34239777

[13] Chen O , Donnelly CR , Ji RR . Regulation of pain by neuro‐immune interactions between macrophages and nociceptor sensory neurons. Curr Opin Neurobiol. 2019;62:17–25. 10.1016/j.conb.2019.11.006 PMC7266706 31809997

[14] Ji RR , Berta T , Nedergaard M . Glia and pain: is chronic pain a gliopathy? Pain. 2013;154(Suppl 1):S10–S28. 10.1016/j.pain.2013.06.022 PMC3858488 23792284

[15] Chen G , Zhang YQ , Qadri YJ , Serhan CN , Ji RR . Microglia in pain: detrimental and protective roles in pathogenesis and resolution of pain. Neuron. 2018;100:1292–311. 10.1016/j.neuron.2018.11.009 PMC6312407 30571942

[16] Flake NM , Hermanstyne TO , Gold MS . Testosterone and estrogen have opposing actions on inflammation‐induced plasma extravasation in the rat temporomandibular joint. Am J Physiol Regul Integr Comp Physiol. 2006; 291:R343–8. 10.1152/ajpregu.00835.2005 16469833

[17] Mapplebeck JCS , Beggs S , Salter MW . Sex differences in pain: a tale of two immune cells. Pain. 2017;158:S43–9. 10.1097/j.pain.0000000000000389 26785152

[18] Classification of Tumours . Head and neck tumours. 5th ed. Lyon, France: World Health Organization; 2022.

[19] Naik K , Janal MN , Chen J , Bandary D , Brar B , Zhang S , et al. The histopathology of oral cancer pain in a mouse model and a human cohort. J Dent Res. 2021;100:194–200. 10.1177/0022034520961020 PMC8173346 33030108

[20] Lisboa MRP , Pereira AF , Alves BWF , Dias DBS , Alves LCV , da Silva CMP , et al. Blockage of the fractalkine pathway reduces hyperalgesia and prevents morphological glial alterations‐comparison between inflammatory and neuropathic orofacial pain in male rats. J Neurosci Res. 2024;102:e25269. 10.1002/jnr.25269 38284851

[21] Young K , Morrison H . Quantifying microglia morphology from photomicrographs of immunohistochemistry prepared tissue using ImageJ. J Vis Exp. 2018;136:57648. 10.3791/57648 PMC6103256 29939190

[22] Bouaoud J , Foy JP , Tortereau A , Michon L , Lavergne V , Gadot N , et al. Early changes in the immune microenvironment of oral potentially malignant disorders reveal an unexpected association of M2 macrophages with oral cancer free survival. Oncoimmunology. 2021;10: 1944554. 10.1080/2162402x.2021.1944554 PMC8238000 34239777

[23] Boutilier AJ , Elsawa SF . Macrophage polarization states in the tumor microenvironment. Int J Mol Sci. 2021;22:6995. 10.3390/ijms22136995 PMC8268869 34209703

[24] Urch CE , Dickenson AH . Neuropathic pain in cancer. Eur J Cancer. 2008;44:1091–6. 10.1016/j.ejca.2008.03.015 18492553

[25] Sessle BJ . Peripheral and central mechanisms of orofacial inflammatory pain. Int Rev Neurobiol. 2011;97:179–206. 10.1016/b978-0-12-385198-7.00007-2 21708311

[26] Pankrath F , Pelai EB , de Oliveira‐Souza AI , Baghbaninaghadehi F , Dennet L , Svensson P , et al. Integration of nociceptive activity from orofacial, cranial and cervical regions in the trigeminocervical nucleus: a scoping review with clinical implications. J Oro Facial Pain Headache. 2011;39:1–12. 10.22514/jofph.2025.042 PMC12531578 41070561

[27] Bai Q , Liu S , Shu H , Tang Y , George S , Dong T , et al. TNFα in the trigeminal nociceptive system is critical for temporomandibular joint. Mol Neurobiol. 2019;56:278–91. 10.1007/s12035-018-1076-y PMC6698364 29696511

[28] Xiao M , Hu Z , Jiang H , Li C , Guo H , Fang W , et al. The expression of netrin‐1 in the MIA‐induced osteoarthritic temporomandibular joint in mice. Sci Rep. 2021;11:15695. 10.1038/s41598-021-95251-9 PMC8333414 34344989

[29] Costa FA , Moreira Neto FL . Satellite glial cells in sensory ganglia: its role in pain, Rev Bras Anestesiol. 2015;65: 73–81. 10.1016/j.bjan.2013.07.013 25497752

[30] Hang LY , Gu Y , Chen Y . Communication between neuronal somata and satellite glial cells in sensory ganglia, Glia. 2013;61:1571–81. 10.1002/glia.22541 PMC3758405 23918214

[31] Karperien A , Ahammer H , Jelinek HF . Quantitating the subtleties of microglial morphology with fractal analysis. Front Cell Neurosci. 2013;7:3. 10.3389/fncel.2013.00003 PMC3558688 23386810

[32] Chen O , Luo X , Ji RR . Macrophages and microglia in inflammation and neuroinflammation underlying different pain states. Med Rev. 2023;3:381–407. 10.1515/mr-2023-0034 PMC10811354 38283253

[33] Fernández‐Arjona MDM , Grondona JM , Fernández‐Llebrez P , López‐Ávalos MD . Microglial morphometric parameters correlate with the expression level of IL‐1β, and allow identifying different activated morphotypes. Front Cell Neurosci. 2019;13:472. 10.3389/fncel.2019.00472 PMC6824358 31708746

[34] Freeman SE , Patil VV , Durham PL . Nitric oxide‐proton stimulation of trigeminal ganglion neurons increases mitogen‐activated protein kinase and phosphatase expression in neurons and satellite glial cells. Neuroscience. 2008;157:542–55. 10.1016/j.neuroscience.2008.09.035 PMC2642960 18938228

[35] Silva Servato JP , Ueira Vieira C , de Faria PR , Cardoso SV , Loyola AM . The importance of inducible nitric oxide synthase and nitrotyrosine as prognostic markers for oral squamous cell carcinoma. J Oral Pathol Med. 2019;48:967–75. 10.1111/jop.12942 31379002

[36] Svensson CI , Medicherla S , Malkmus S , Jiang Y , Ma JY , Kerr I , et al. Role of p38 mitogen activated protein kinase in a model of osteosarcoma‐induced pain. Pharmacol Biochem Behav. 2008;90:664–75. 10.1016/j.pbb.2008.05.016 18584857

