# Supplementary material for: Neuroimmune alterations and nociceptive behavior in oral carcinogenesis in rats
Source: Eur J Oral Sci. 2026 May 14;134(5):e70107. doi: 10.1111/eos.70107 (PMC13584683; doi:10.1111/eos.70107)
Supplement: Supplementary file 1 [file EOS-134-e70107-s002.docx]

Table S1- Analysis of facial expressions in rats underwent oral carcinogenesis

|  | 0W | 4w | 8w | 12w | 16w | 20w |
| --- | --- | --- | --- | --- | --- | --- |
| control | 0 (0-0) | 0 (0-0) | 0 (0-0) | 0 (0-1) | 0 (0-0) | 0 (0-1) |
| 4-nqo | 0 (0-0) | 0 (0-1) | 0 (0-1) | 0 (0-3) | 0 (0-2) | 2 (0-3) |
| *p* | >0,9999 | >0,9999 | >0,9999 | >0,9999 | >0,9999 | 0,0023 |

The sum of the scores for orbital pinching, nose flattening, and ear changes was expressed as median (minimum–maximum). 4- NQO= 4-Nitroquinoline 1-oxide. 0W= baseline time. 4W= 4-NQO applied to the tongue (2×/day) for 4 weeks. 8W = 4-NQO applied to the tongue (2×/day) for 8 weeks; 12W = 4-NQO (2×/day) for 12 weeks; 16W = 4-NQO (2×/day) for 16 weeks; 20W = 4-NQO (2×/day) for 20 weeks. Mann-Whitney test
