# Supplementary material for: Neuroimmune alterations and nociceptive behavior in oral carcinogenesis in rats
Source: Eur J Oral Sci. 2026 May 14;134(5):e70107. doi: 10.1111/eos.70107 (PMC13584683; doi:10.1111/eos.70107)
Supplement: Supplementary file 2 [file EOS-134-e70107-s003.docx]

**Table S2-** Temporal distribution of histopathological alterations in the tongue during 4-NQO exposure

Control= no exposure to 4-NQO). 4-NQO-8W, 4-NQO-12W, 4-NQO-16W, and 4-NQO-20W = exposed to 4-NQO for 8, 12, 16, or 20 weeks, respectively.

|  | CONTROL | 4-NQO-8W | 4-NQO-12W | 4-NQO-16W | 4-NQO-20W |
| --- | --- | --- | --- | --- | --- |
| *Normal epithelium* | 6 (100%) |  |  |  |  |
| *Hyperkeratosis without dysplasia* |  |  | 2 (25%) |  |  |
| *Mild dysplasia (with hyperkeratosis)* |  | 5 (62,5%) | 6 (75%) | 3 (37,5%) | 2 (20%) |
| *Moderate dysplasia* |  | 2 (25%) |  | 2 (25%) | 1 (10%) |
| *Severe dysplasia* |  | 1 (12,5%) |  | 2 (25%) | 2 (20%) |
| *OSCC* |  |  |  | 1 (12,5%) | 4 (40%) |
| *Microinvasive oSCC* |  |  |  |  | 1 (10%) |
