# Supplementary material for: Neuroimmune alterations and nociceptive behavior in oral carcinogenesis in rats
Source: Eur J Oral Sci. 2026 May 14;134(5):e70107. doi: 10.1111/eos.70107 (PMC13584683; doi:10.1111/eos.70107)
Supplement: Supplementary file 3 [file EOS-134-e70107-s005.docx]

|  | LOCALIZATION | | papillary | Invasive |  |  | deeply invasive | multiple | single |
| --- | --- | --- | --- | --- | --- | --- | --- | --- | --- |
| 4-NQO-16W | | Posterior dorsal third (100%) | __ | __ | (1) | (2) | (3) 100% | __ | (1) 100% |
| 4-NQO-20W | | Posterior dorsal third (100%) | 5 (100%) | __ |  |  | __ | 3 (60%) | 2 (40%) |

**Table S3.** Macroscopic characterization and anatomical distribution of tongue oral cancer

lesions at 16 and 20 weeks of 4-NQO exposure.
